# Global integration, local constraints: how internationalized scientific development shapes early-career research in Latin America

**DOI:** 10.3389/fsoc.2026.1807485

**Published:** 2026-05-11

**Authors:** Kevin Aguirre-Carvajal, Vinicio Armijos-Jaramillo

**Affiliations:** 1 Bio-Cheminformatics Research Group, Universidad de Las Américas, Quito, Ecuador; 2 Carrera de Ingeniería en Biotecnología, Facultad de Ingeniería y Ciencias Aplicadas, Universidad de Las Américas, Quito, Ecuador

**Keywords:** academic labor conditions, early-career researchers, global scientific inequalities, internationalization of science, Latin America

## Abstract

Scientific development in Latin America has expanded through intensified international collaboration, yet structural asymmetries in global knowledge production shape how these gains translate into local research capacity. Early-career researchers (ECRs) occupy a central role in sustaining scientific output, navigating internationally defined expectations of productivity, mobility and visibility while facing precarious employment and limited institutional support. This perspective examines how internationalization pathways, performance metrics and labor structures interact to reproduce dependency within local research systems, highlighting tensions between global integration and sustainable academic careers. We argue that addressing these challenges requires coordinated policy and institutional responses, including public investment, strengthened domestic doctoral programs, transparent career pathways and alignment of international collaboration with local priorities. By centering the experiences of ECRs, this work provides a lens for understanding the broader implications of internationalized scientific development for research capacity, equity and policy in the Global South.

## 1 Introduction

Scientific development does not occur under symmetrical conditions globally. Countries in the Global South continue to face structural disadvantages that constrain their full participation in international knowledge production, including historically low investment in research and development, fluctuating science policies and limited access to advanced training programs (Dakhil et al., 2024; Vessuri et al., 2014). These asymmetries shape not only patterns of scientific output but also institutional capacity to sustain research careers and knowledge production over time.

These structural asymmetries are reflected in disparities in research and development investment across global regions. Countries in Latin America invest less than 1% of their gross domestic product (GDP), with notable exceptions such as Brazil, which approaches but still remains below levels observed in leading scientific economies. In contrast, countries such as Israel and South Korea invest more than 5% of GDP, while major economies including the United States, Germany and Japan maintain investment levels around 3% (RICYT, 2024).

Within this uneven landscape, Latin America has experienced a notable expansion of 40% in scientific production over the past decade (RICYT, 2024). Between 2009 and 2014, publication output increased steadily, driven primarily by Mexico, Brazil, Colombia, Chile and Argentina. This trend accelerated in recent years, with countries that had previously been marginal in regional science—such as Ecuador, Peru and Uruguay—significantly expanding their contributions (Belli and Baltà, 2019; Soler, 2021). These developments are frequently interpreted as evidence of convergence with global scientific systems and deeper integration into international research networks.

This growth has been supported by a combination of factors, including targeted investments in research infrastructure, the expansion of international collaborations and the strengthening of local research networks (Carrasco-Ahen et al., 2025). However, these transformations have unfolded unevenly across the region. While countries such as Mexico, Brazil, Argentina, Chile, Colombia and Costa Rica have consolidated research capacity and doctoral training infrastructures, others—including Bolivia, Panama and Guatemala—continue to face persistent challenges related to limited state funding, administrative instability and scarce local doctoral programs (Huete-Perez and Salvatierra, 2025; Lopez-Verges et al., 2021). As a result, aggregate gains in scientific productivity coexist with substantial institutional fragility.

This divergence raises a central tension in contemporary analyses of Latin American science: to what extent does increased publication output reflect genuine qualitative autonomy, rather than expanded participation within externally structured research systems? And how do these dynamics shape the working conditions, expectations and career trajectories of those entering the academic profession?

The limits of internationally driven models of scientific development in Latin America become particularly visible when examined through the position of early-career researchers (ECRs)—here understood as doctoral candidates, postdoctoral researchers and scholars in the initial stages of their academic careers (Lopez-Verges et al., 2021). ECRs occupy a structurally central position within contemporary research systems: they constitute a large share of the scientific workforce, sustain day-to-day research production and serve as key vectors of methodological innovation, disciplinary renewal and emerging research practices. Compared to more established academics, ECRs tend to exhibit greater demographic and epistemic diversity, drawing on varied socioeconomic backgrounds, institutional trajectories and national contexts. At the same time, their strong engagement with new tools, methods and collaborative practices—often coupled with extended time investment in research—coincides with heightened exposure to institutional instability and labor precarity (Kent et al., 2022).

Because of their key role in sustaining scientific production and renewal, ECRs are positioned at the entry point of professional research trajectories, where they are required to internalize globally standardized expectations of productivity and scientific visibility that increasingly define academic success (Cejas et al., 2025). These demands are often pursued under institutionally constrained conditions, producing a misalignment between global evaluative standards and local research environments. This misalignment is most acutely experienced at the early-career stage, where researchers must navigate high performance expectations amid structural limitations that shape access to training, funding and career stability (Ciarli and Ràfols, 2019).

Examining early-career trajectories therefore offers a strategic lens for understanding how internationally driven models of scientific development operate in practice in Latin America and where their limitations emerge. To understand why recent growth in scientific output does not consistently translate into stable academic development, it is necessary to examine how this growth is produced—specifically, how it is sustained through the labor, mobility and career trajectories of ECRs.

## 2 Global north–centered standards and research dependency

Examining internationally driven models of scientific development requires attention to the structural hierarchies that organize global knowledge production. In Latin America, these hierarchies generate multiple forms of dependency that shape not only institutional research agendas but also academic career trajectories. These dependencies include reliance on externally defined research priorities, evaluation standards, funding sources and professional benchmarks anchored primarily in institutions based in the Global North (Castro Torres and Alburez-Gutierrez, 2022; Locke, 2014; Naidu et al., 2024). While such conditions affect research systems as a whole, their consequences are unevenly distributed across career stages, with early phases of academic trajectories being particularly exposed to externally defined expectations.

A key mechanism sustaining these dependencies is the unequal control over research priorities. Research topics, theoretical frameworks and methodological standards are frequently shaped by institutions and funding agencies based in the Global North, influencing which questions are considered legitimate, which forms of knowledge are deemed publishable and the volume and venues of publication required for institutional recognition (Ciarli and Ràfols, 2019; Khisa et al., 2024; Oliveira and Pinto, 2024). ECRs in Latin America therefore often align their work with externally defined agendas to secure funding and career advancement, even when these priorities are weakly aligned with local scientific needs.

These dependencies are further reinforced by evaluation regimes based on standardized performance indicators that convert externally defined research priorities into criteria of academic success. International rankings, publication counts, impact factors and citation metrics increasingly shape institutional strategies and early career trajectories, where progression depends on narrowly defined benchmarks (Locke, 2014; López, 2019; Moher et al., 2018).

Although framed as objective measures of excellence, these indicators disproportionately reward access to high-impact journals, international networks and English-language publishing infrastructures—resources that remain disparately distributed across the global academic system. As a result, ECRs operating within Latin American institutions face a structural misalignment between international performance standards and the institutional conditions under which research is produced (Cejas et al., 2025; Chen et al., 2025).

Within this evaluative framework, the normalization of publish-or-perish cultures functions as a disciplinary mechanism that intensifies pressures on ECRs to prioritize continuous publication in internationally indexed journals. Compliance with these expectations often comes at the expense of contextually grounded or locally relevant research agendas, which are less visible within dominant evaluation systems (Arellano-Rojas et al., 2022; Liu et al., 2024; van Dalen and Henkens, 2012). Consequently, increases in scientific output may coexist with declining institutional autonomy, as research agendas, publication strategies and career evaluations remain externally anchored.

These structural conditions configure how international collaboration is organized and help explain why early-career researchers emerge as primary risk bearers within contemporary models of scientific internationalization.

## 3 Internationalization pathways and early-career researchers

Within the hierarchically structured global scientific system described above, international collaboration has emerged as a central pathway through which Latin American research systems expand their scientific output. Mobility programs, co-supervised doctoral degrees and international research networks function as key mechanisms through which researchers access advanced training, research infrastructure and publishing opportunities often unavailable within local institutions (Echeverría-King et al., 2023; Lima-Toivanen et al., 2025). Crucially, these internationalization mechanisms are institutionally structured around researchers in early stages of their careers.

Programs such as Brazil’s CAPES sandwich doctoral scholarships, Mexico’s CONACYT fellowships abroad and European Erasmus+ mobility schemes explicitly target ECRs, facilitating international research stays, joint degrees and co-supervision arrangements (Saling, 2025; Souza and Pertusatti, 2024; Żebryk et al., 2021). Promising ECRs frequently pursue doctoral training abroad, particularly in European systems such as Spain and Germany (Belli and Morín Nenoff, 2022; Lima-Toivanen et al., 2025).

International mobility enhances both human and social capital, strengthening methodological expertise, collaborative networks and research productivity (Momeni et al., 2022; Soler, 2021). Beyond individual skill acquisition, internationally trained ECRs play a structural role in linking local research environments to global academic practices by facilitating the circulation of methods, collaborative norms and research standards across institutional and national boundaries (Djaafara et al., 2025; Ito et al., 2025; Lescano et al., 2019).

At the same time, positioning ECRs at the operational core of international collaboration exposes them to heightened professional risk. Mobility schemes and co-supervised doctoral arrangements often rely on temporary contracts, geographic displacement and uncertain reintegration into home institutions. While these pathways enhance skills and visibility, they also relocate the costs and risks of internationalization from institutions onto individuals (Montoya, 2021; Souza and Pertusatti, 2024; Żebryk et al., 2021). Thus, the mechanisms driving scientific growth simultaneously reproduce forms of dependency by reconfiguring early-career employment trajectories under conditions of labor uncertainty and precarity.

## 4 Employment implications for early-career researchers

From a labor perspective, internationally driven models of scientific development generate deeply ambiguous outcomes for ECRs. While international training and collaboration enhance individual competitiveness and scientific visibility, they do not reliably translate into stable academic employment upon return to local institutions. Instead, many ECRs confront conditions of overqualification, in which advanced credentials exceed the opportunities available within domestic academic labor markets (Castro et al., 2024; O’Brady et al., 2025).

In Latin America, labor-market studies show that roughly 70% of young workers under 29 years old occupy precarious positions, characterized by informal or non-standard contracts and limited social protection (Florez-Vaquiro and Hincapié-Aldana, 2025). Within academic systems, these conditions are mirrored in employment structures dominated by temporary contracts, postdoctoral fellowships and externally funded positions, with limited transition to permanent roles (Pineda, 2023). These structural conditions are further intensified for ECRs navigating academic labor markets.

For example, in Mexico, studies of doctoral-program graduates show that many PhDs end up teaching at undergraduate levels or in administrative roles, rather than securing permanent academic positions (Gonzalez, 2018). A similar pattern can be observed in Paraguay, where recent evidence on PhD holders’ career trajectories highlights structural constraints in academic employment pathways. Although the majority of doctoral graduates remain within academia, more than half of ECRs hold multiple jobs simultaneously (Villalba and Badía, 2025). This pattern suggests a fragmented and unstable labor market, in which highly trained researchers must combine multiple positions to achieve economic and professional stability, reflecting a structural mismatch between advanced training and the availability of stable academic employment.

This mismatch is compounded by the widespread reliance on short-term and insecure contracts. ECRs often navigate a succession of temporary research appointments, adjunct teaching positions or externally funded fellowships, accompanied by sustained pressure to publish in international journals (Herschberg et al., 2018; SSM ECR Subcommittee and Hayes, 2023; van Dalen and Henkens, 2012). Such arrangements offer limited job security, restricted access to institutional resources and few clear pathways to permanent positions, particularly in systems characterized by constrained public funding.

In parallel, teaching overload further undermines research continuity (Ramirez-Montoya et al., 2023). Heavy teaching and administrative responsibilities—frequently assigned to compensate for staffing shortages—reduce time available for research, despite continued evaluation based on productivity metrics aligned with high-output academic systems. This contradiction intensifies workload pressures while narrowing the conditions under which ECRs can meet internationally defined expectations (Osare and Keshvari, 2024; Sandmeier et al., 2022).

At the institutional level, these dynamics produce a persistent gap between symbolic benefit and material support. Universities may gain reputational advantages from international collaborations and increased publication output while failing to provide stable employment conditions or long-term career prospects for the researchers who sustain these activities (Castro et al., 2024; Roll et al., 2025). As a result, ECRs increasingly absorb the risks of internationally driven scientific development, with implications for individual well-being and long-term research capacity. In some cases, prolonged precarity leads highly trained researchers to exit academic careers or seek permanent employment abroad, further weakening local research systems.

Taken together, these patterns indicate that employment precarity among ECRs is structurally embedded in prevailing models of internationalized scientific development, underscoring the need for coordinated policy and institutional responses.

## 5 Institutional actions to support research capacity and early-career researchers

The dynamics analyzed in the preceding sections—namely the externalization of research agendas, the standardization of evaluation regimes, the internationalization of doctoral training and the transfer of labor risks onto early-career researchers—have direct implications for research policy and institutional capacity-building in Latin America. Responding to these linked forms of dependency requires shifting focus from individual adaptation to collective institutional and policy strategies (Echeverría-King et al., 2023).

Among the institutional and policy responses required, sustained public investment in science constitutes a foundational condition for reducing structural dependency, given its long-established association with long-term economic and technological development (Coccia, 2008). Indeed, studies on development and innovation indicate that the returns on scientific investment can be particularly high in low- and middle-income countries, where research systems remain underdeveloped (Jaffe et al., 2013; Moyo and Phiri, 2024). In this context, additional investment has the potential to unlock latent scientific capacity, expand training opportunities and generate substantial gains in productivity and innovation. However, investment alone is not sufficient: realizing the broader benefits of science requires institutional mechanisms that ensure research outputs are effectively translated into economic growth and sustainable development.

Achieving this translation depends on the coordination of multiple institutional actors. Effective research systems require alignment between public policy, university governance, private-sector engagement and labor-market structures (Abramo and D’Angelo, 2024). In the absence of institutional mechanisms that provide stable career pathways and adequate working conditions, the benefits of scientific investment remain difficult to realize. Early-career researchers are important in this process: they constitute the primary workforce of contemporary research systems and represent the main conduit through which training, innovation and productivity are transformed into long-term institutional capacity.

An illustrative example of strategic investment can be observed in the Science for Africa Foundation, which has emphasized the importance of targeted funding approaches to strengthen regional research systems. In 2023, the foundation launched From Science to Impact initiative, a five-year program designed to support research in priority areas such as health, agriculture, climate and environmental sustainability. To date, the program has supported more than 400 African research leaders across 33 countries and funded 14 multi-institutional consortia focused on health and climate-related challenges. This initiative demonstrates how coordinated and strategically allocated investment can expand research capacity, foster regional collaboration and enhance the societal relevance of scientific production (Science for Africa Foundation, 2024).

For Latin American countries, insufficient funding represents a missed opportunity. Without stable investment, internationally trained ECRs are more likely to seek opportunities in developed countries, contributing to brain drain and weakening local scientific ecosystems (Lee et al., 2025). This can be detrimental to maintaining a strong scientific system that is important to exchange the investment into economical growth.

A key priority is the strengthening of local doctoral programs and research infrastructure in response to the reliance on foreign training pathways identified earlier. Expanding the availability and quality of domestic doctoral training is essential to reduce dependence on foreign training pathways and to consolidate endogenous research capacity. Strong local doctoral systems enable institutions to retain talent, cultivate locally relevant research agendas and sustain scientific communities over time (Celis et al., 2025; Chen et al., 2025).

Beyond training and funding, academic labor governance represents a critical site of intervention. The expansion of internationally trained ECRs has not been matched by the development of stable and transparent academic career pathways. Policies promoting fair contracts, predictable career progression and competitive remuneration are central to addressing the labor precarity generated by internationalized training systems (Altbach, 2007; Hawcroft et al., 2023; Miranda-Nieto et al., 2022).

Some national initiatives provide insight into these dynamics. For example, programs such as Brazil’s CAPES and Mexico’s CONACYT have significantly expanded doctoral training and international mobility opportunities, representing important advances in scientific capacity building. However, these initiatives have also been criticized for insufficient reintegration mechanisms for returning researchers, contributing to employment instability and limited absorption into domestic academic systems (Arce Miyaki and Gomis Hernández, 2019; Lopez-Verges et al., 2021; Malafaia et al., 2024). As such, they illustrate both the potential and the limitations of internationalization policies, particularly when expansion of training is not matched by the development of stable local career structures.

A contrasting case can be observed in Ecuador’s SENESCYT scholarship program. Evidence indicates that by 2016, more than 4,800 postgraduate scholarship recipients had returned to the country, with 97.6% complying with reintegration requirements, suggesting a relatively strong capacity to facilitate return migration and mitigate brain drain (Hitner and Tapia López, 2018). In addition, scholarship holders exhibited approximately 9% higher employment income compared to individuals who pursued postgraduate training locally, pointing to potential individual-level benefits associated with international training pathways (Ponce and Cedeño, 2021).

However, these benefits do not appear to be immediate: positive career effects tend to emerge only after several years following reintegration. Moreover, employment outcomes reveal structural constraints, as the majority of returnees are absorbed into academic institutions, with limited participation in the private sector, and a substantial share occupying temporary positions or roles not directly linked to research activities (Hitner and Tapia López, 2018). These patterns suggest that, while return migration has been effectively achieved, important gaps remain between the expansion of advanced training and the capacity of domestic systems to provide stable and research-oriented career opportunities.

From this perspective, policies that strengthen local funding structures, improve employment stability and align international collaboration with domestic research priorities are not peripheral but central to scientific development. Without deliberate efforts to retain and support ECRs, internationally driven models of scientific development risk reinforcing dependency rather than fostering sustainable and inclusive growth in Latin America.

## 6 Conclusion

Over the past decade, scientific production in Latin America has expanded substantially, driven by intensified international collaboration and the growing participation of ECRs in global academic networks. However, this growth has unfolded within a global scientific system structured by enduring asymmetries in agenda-setting, evaluation and academic labor organization. As a result, gains in publication output and visibility do not automatically translate into institutional autonomy, stable research careers, or sustained local scientific capacity.

ECRs occupy a pivotal yet vulnerable position within this system. Their labor underpins much of the region’s scientific growth, while they simultaneously bear the risks generated by internationally driven models of development. Addressing these tensions requires not abandoning international collaboration, but restructuring how it is embedded within local academic systems. Strengthened doctoral programs, stable career pathways, aligned collaboration strategies and retention-focused policies are essential for building a more equitable and resilient scientific system in Latin America.

As early-career researchers based in Latin America, we recognize that the dynamics described in this perspective are not only structural but also experiential. Our own academic trajectories have been shaped by the pressures of international publication standards, the prioritization of indexed journals, and the need to align research agendas with globally recognized frameworks. These expectations have influenced decisions regarding research topics, publication strategies, and collaboration pathways, often requiring a balance between locally relevant questions and internationally visible outputs.

At the same time, our position within this system is not purely passive. While we are subject to its constraints, we also actively navigate and reproduce some of its logics through participation in international collaborations and adherence to evaluation metrics. This dual position—both as subjects shaped by the system and as agents operating within it—highlights the complexity of transforming scientific development pathways. It also shapes the production of this manuscript itself, including choices about framing, language, and publication strategy, which are inevitably influenced by the same structural conditions we critically examine. Recognizing this positionality is essential for understanding how structural inequalities are experienced, negotiated, and potentially challenged at the level of early-career researchers.

## Data Availability

The original contributions presented in the study are included in the article/supplementary material, further inquiries can be directed to the corresponding author.
